# Multiparametric mapping by cardiovascular magnetic resonance imaging in cardiac tumors

**DOI:** 10.1186/s12968-023-00938-9

**Published:** 2023-06-22

**Authors:** Pengfei Yue, Ziqian Xu, Ke Wan, Yinxi Tan, Yuanwei Xu, Xiaotong Xie, David Mui, Cheng Yi, Yuchi Han, Yucheng Chen

**Affiliations:** 1 Division of Abdominal Tumor Multimodality Treatment, Cancer Center, West China Hospital, Sichuan University, Chengdu, Sichuan China; 2 Department of Cardiology, West China Hospital, Sichuan University, Chengdu, Sichuan 610041 People’s Republic of China; 3 Department of Radiology, West China Hospital, Sichuan University, Chengdu, Sichuan China; 4 Department of Geriatrics, West China Hospital, Sichuan University, Chengdu, Sichuan China; 5 West China School of Public Health, Sichuan University, Chengdu, China; 6 Perelman School of Medicine, University of Pennsylvania, Philadelphia, PA USA; 7 Cardiovascular Division, Wexner Medical Center, The Ohio State University, Columbus, OH USA

**Keywords:** Cardiac tumor, Benign, Primary malignant, Cardiac magnetic resonance, Mapping

## Abstract

**Background:**

There is a paucity of quantitative measurements of cardiac tumors and myocardium using parametric mapping techniques. This study aims to explore quantitative characteristics and diagnostic performance of native T1, T2, and extracellular volume (ECV) values of cardiac tumors and left ventricular (LV) myocardium.

**Methods:**

Patients with suspected cardiac tumors who underwent cardiovascular magnetic resonance (CMR) between November 2013 and March 2021 were prospectively enrolled. The diagnoses of primary benign or malignant tumors were based on pathologic findings if available, comprehensive medical history evaluations, imaging, and long-term follow-up data. Patients with pseudo-tumors, cardiac metastasis, primary cardiac diseases, and prior radiotherapy or chemotherapy were excluded. Multiparametric mapping values were measured on both cardiac tumors and the LV myocardium. Statistical analyses were performed using independent-samples *t-test*, receiver operating characteristic, and Bland–Altman analyses.

**Results:**

A total of 80 patients diagnosed with benign (n = 54), or primary malignant cardiac tumors (n = 26), and 50 age and sex-matched healthy volunteers were included. Intergroup differences in the T1 and T2 values of cardiac tumors were not significant, however, patients with primary malignant cardiac tumors showed significantly higher mean myocardial T1 values (1360 ± 61.4 ms) compared with patients with benign tumors (1259.7 ± 46.2 ms), and normal controls (1206 ± 44.0 ms, all *P* < 0.05) at 3 T. Patients with primary malignant cardiac tumors also showed significantly higher mean ECV (34.6 ± 5.2%) compared with patients with benign (30.0 ± 2.5%) tumors, and normal controls (27.3 ± 3.0%, all *P* < 0.05). For the differentiation between primary malignant and benign cardiac tumors, the mean myocardial native T1 value showed the highest efficacy (AUC: 0.919, cutoff value: 1300 ms) compared with mean ECV (AUC: 0.817) and T2 (AUC: 0.619) values.

**Conclusion:**

Native T1 and T2 of cardiac tumors showed high heterogeneity, while myocardial native T1 values in primary malignant cardiac tumors were elevated compared to patients with benign cardiac tumors, which may serve as a new imaging marker for primary malignant cardiac tumors.

**Supplementary Information:**

The online version contains supplementary material available at 10.1186/s12968-023-00938-9.

## Introduction

Cardiac tumors are rare and are found in 0.002–0.003% in large autopsy studies [1–3]. Benign cardiac tumors account for approximately three quarters of primary cardiac tumors, with myxoma and rhabdomyoma being the most common tumors in adults and children, respectively [2, 4]. Primary malignant cardiac tumors are extremely rare [5]. The identification of benign and malignant cardiac mass by non-invasive imaging methods is of great significance for clinical treatment decisions and patient prognosis [6–9]. With the advantage of large field of view, superior tissue contrast, and the ability to evaluate tissue characteristics, CMR has been regarded as a versatile and powerful tool in differentiating benign and malignant tumors [10–13]. Cine images with high spatial and temporal resolution provide detailed morphological and functional information. Traditional T1 and T2 weighted images (with or without fat suppression) provide qualitative information on mass composition, however, T2 weighted images are susceptible to motion artifacts, slow flow, and signal intensity variability [14]. First pass perfusion evaluates tumor vascularity, which can help to differentiate between benign and malignant tumors [15]. The presence and pattern of late gadolinium enhancement (LGE) in the tumor could represent vascularity and necrosis and has been shown to be able to differentiate neoplasm from thrombus, as well as to predict survival [16]. Myocardial LGE can identify focal fibrotic components but is insensitive to diffuse fibrosis. Parametric mapping techniques have been studied extensively in cardiomyopathy, while their value in the evaluation of tumors and myocardium in patients with cardiac mass has not been previously studied.

The effects of cardiac tumors on the myocardium may help differentiating benign and malignant tumors. In a previous study that included 622 autopsies of patients with malignant neoplasms, patients with cardiac involvement were found to have a significantly higher incidence of ECG abnormalities and arrhythmia compred to patients without cardiac involvement [17]. Benign tumors can have expansive growth and compress surrounding tissues, but they are usually separated from the surrounding by a fibrous capsule [18]. Malignant tumors generally present as invasive growth or local infiltration with the interaction of surrounding tissues, which may cause inflammatory changes and activate fibroblasts [18, 19]. The potential pathophysiologic changes in the myocardium caused by cardiac tumors remain unclear. However, few studies discuss the difference of cardiac function and myocardial tissue characteristics in patients with benign and malignant cardiac tumors. CMR multi-parametric mapping allows the quantitative evaluation of myocardial tissue characteristics [20]. Native T1 and ECV values are correlated with diffuse interstitial fibrosis [21, 22]. T2 mapping has the potential for application in myocardial inflammation and edema assessment [23].

Thus, we hypothesized that quantitative multi-parametric mapping (T1, T2, and ECV) may help to elucidate the tissue characteristics of cardiac tumors, as well as the potential effect of cardiac tumors on the LV myocardium. The study was aimed at exploring the tissue characteristics of cardiac tumors and the potential effect of different types of cardiac tumors on myocardium by T1, T2, and ECV values between patients with benign cardiac tumors and primary malignant cardiac tumors.

## Methods

### Study population and study design

Patients who were referred for CMR between November 2013 and March 2021 with suspected cardiac tumors were recruited. This prospective single-center registered study was approved by the ethics committee of West China Hospital. All procedures followed the Declaration of Helsinki. All participants provided written informed consent for study participation.

**Fig. 1:**
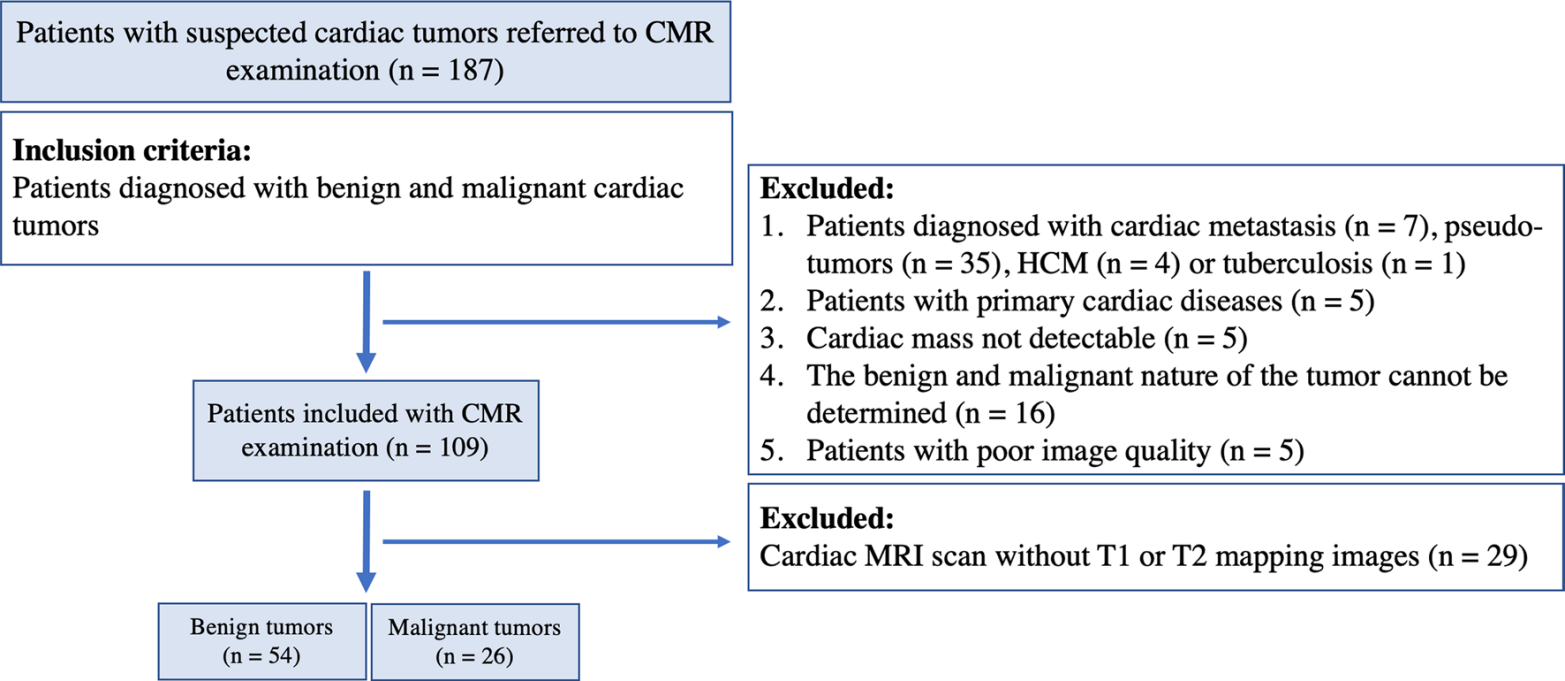
The study flow-chart of patients with primary cardiac tumors

Since the onset of cardiac tumors has a wide-age range, and different types of tumors have diverse predilection in terms of age, for example, rhabdomyomas are primarily found in childhood, we included patients of all age groups to make the study more comprehensive and representative [4]. The inclusion criteria were primary benign and malignant cardiac tumors. Among patients who underwent surgical resection or biopsy, the diagnoses were based on pathological results. For those who did not, the final diagnosis was made based on comprehensive evaluations of medical history, clinical symptoms, CMR, positron emission tomography-computed tomography (PET-CT) results, and long-term follow-up of more than 5 years [9, 24, 25]. Exclusion criteria were patients with cardiac pseudo-tumors such as thrombus, cyst, cardiac echinococcosis, leaflet vegetation, or tuberculosis, as well as hypertrophic cardiomyopathy or aneurysms misdiagnosed as tumors. Cardiac thrombus were diagnosed based on the classic criteria [12]. Thrombus does not have contrast uptake and therefore appears dark on LGE, while the surrounding area may show high uptake, which can be distinguished from the tumor [8]. In order to study the effect of primary malignant cardiac tumor on the myocardium, patients with cardiac metastasis were also excluded (n = 7). These patients had a clear history of extracardiac primary tumor and pathological confirmation. We also excluded patients with primary cardiac diseases including known coronary artery disease, primary cardiomyopathy, patients with no detectable mass on CMR, history of prior chemotherapy or radiotherapy, or poor image quality. The inclusion and exclusion workflow chart of patients is presented in Fig. 1. We also included 50 age- and sex-matched normal controls from a previous study cohort for comparison [26].

### CMR protocol

CMR examinations were performed at 3T (MAGNETOM Trio or Skyra, Siemens Healthineers, Erlangen, Germany) with breath-holding and ECG gating. The CMR protocol included a standard sequence of balanced steady-state free precession (bSSFP), T1 mapping before and 10–15 min after contrast injection, T2 mapping, and late gadolinium enhancement (LGE). The bSSFP cine images were acquired on consecutive short-axis views and 2-, 3-, and 4-chambers long-axis views. The T1 and T2 mapping images were acquired in three short-axis views (basal, mid, and apical levels) and three long-axis views. Tumor-focused views and sequences were obtained as needed. The typical scan parameters are shown in Additional file 1.

### CMR analyses

We analyzed CMR studies using Medis suite (version 3.2; Medis, Leiden, the Netherlands). Left ventricular (LV) and right ventricular (RV) volumes, ejection fraction (EF), and LV mass were assessed based on consecutive short-axis images according to the standardized protocol of the Society of Cardiovascular Magnetic Resonance (SCMR) post-processing guidelines [27].

**Fig. 2:**
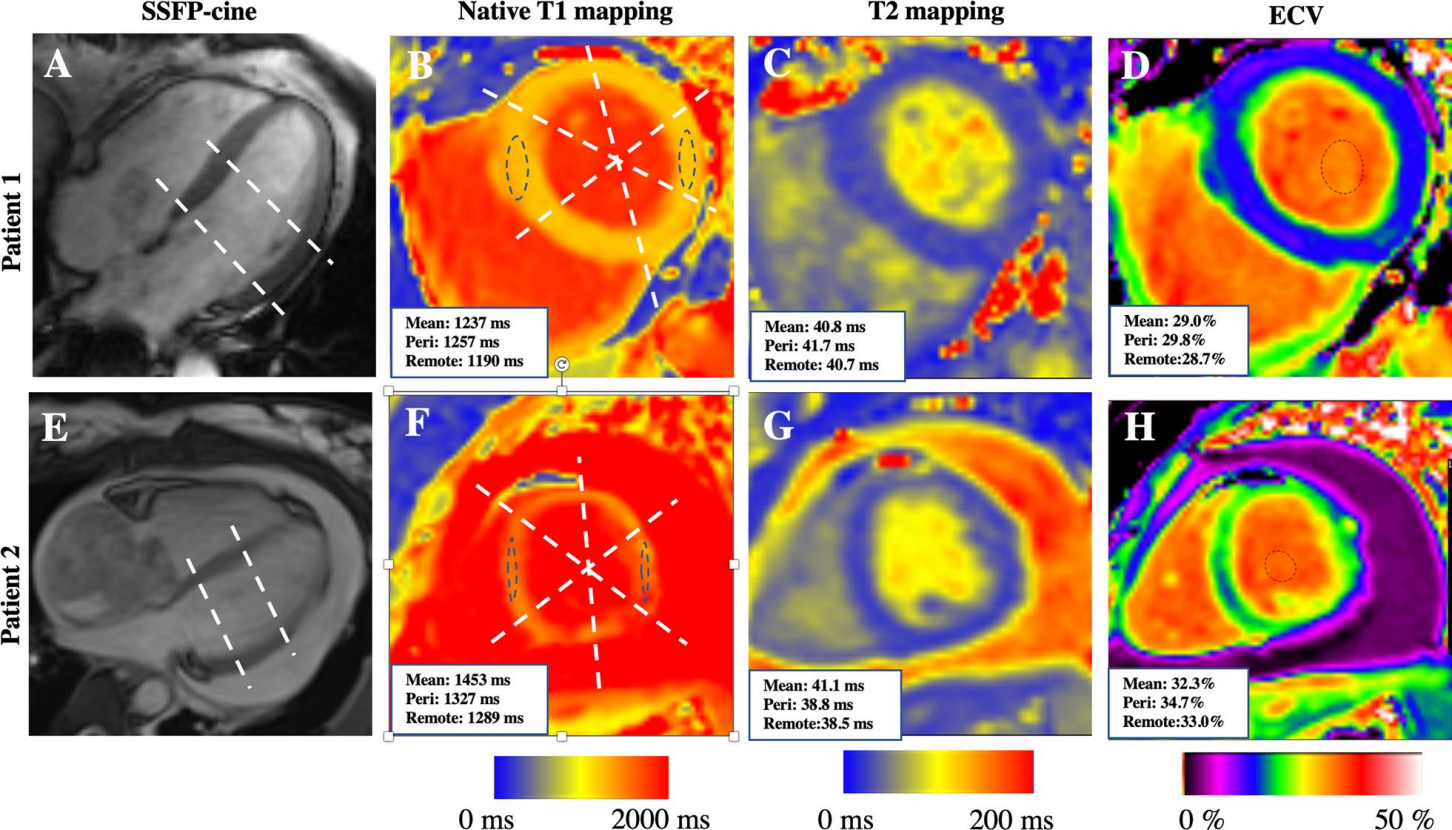
Illustrative images of SSFP-cine, native T1 mapping, T2 mapping, and ECV images of patients with benign and primary malignant cardiac tumors. Patient 1: Patient was diagnosed with benign myxoma. Patient 2: patient was diagnosed with primary malignant tumor. **A**, **E** The 4-chamber view of SFPP-cine image. Dashed lines indicate the basal and mid-ventricular level; **B**, **C**, **F**–**H** short-axis views at mid-ventricular level of mapping images. In T1-mapping images, the dashed lines represent the division of 6 segments on short axis images. *b-SSFP* balanced steady-state free precession, *ECV* extracellular volume

The boundary of cardiac tumors was manually delineated on mapping images with region of interest (ROI) containing more than 20 pixels [27]. The myocardial native T1, T2, and ECV values were measured as the average of basal and mid-levels short-axis views. The endo- and epicardial contours were manually delineated avoiding endocardial trabeculations, epicardial fat, and tumor if present. The segments at basal and mid-ventricular level were subdivided according to the AHA 17-segments model [28, 29]. In patients with tumors located in the atrium, the peri-tumor area was selected to be the segment closest to the tumor at the basal-ventricular slice, while the remote-tumor area was selected on the mid-ventricular slice with the segment from the opposite side. In patients with tumors located in the ventricles, the peri-tumor area was defined as the closest segment next to the mass on the slice closest to the tumor, while the remote-tumor area was defined as the segment on the opposite side of the mass on the most remote slice. The ECV was calculated based on the T1 values before and after contrast injection according to the following formula: (1-hematocrit) (1/T1myo post − 1/T1myo pre)/(1/T1blood post − 1/T1blood pre). Illustrative images of bSSFP cine, native T1 mapping, T2 mapping, and ECV in patients with different cardiac tumors are shown in Fig. 2.

Characteristics of cardiac tumors, including the size, location, mobility, border, and invasiveness were recorded. We also classified the mass location as intra-cavitary or intramural depending on whether the mass was predominantly localized inside the cardiac chambers or invading into the myocardium [30]. Tumor characteristics were evaluated by two investigators (P.F.Y and Z.Q.X, with 4 and 7 years of CMR experience, respectively) blinded to clinical information. The intra- and inter-observer reproducibilities of the measurement of multi-parametric values of cardiac tumors and myocardium were evaluated in 30 randomly selected participants.

### Laboratory biomarkers

Hematocrit and serum cardiac troponin T (cTnT), N-terminal fragment of prohormone brain natriuretic peptide (NT-ProBNP), and creatinine levels were obtained within 3 days of CMR; and tumor biomarkers including alpha-fetoprotein (AFP), carcinoembryonic antigen (CEA), cancer antigen 19-9 (CA19-9), and CA125 were obtained within 1 month of the CMR examination.

### Statistical analysis

Participants were grouped as having a benign cardiac tumor or primary malignant cardiac tumor. Depending on the normality of the distribution, continuous variables were summarized as means (± standard derivation) or medians (with interquartile range [IQR]) and compared by the independent-sample t-test or Mann–Whitney U test. Categorical variables are presented as numbers and percentages and were compared using the Chi-square test or Fisher’s exact test. Receiver operator characteristic (ROC) analysis was performed and the optimal cutoff values were calculated by the Youden index. The intra- and inter-observer variability were assessed by using Bland–Altman analysis, coefficients of variation (CoV), and intra-class correlation coefficients (ICC). Two-tailed *P* < 0.05 was considered statistically significant. Statistical analysis was performed using SPSS 26.0 (IBM, Armonk, New York).

## Results

### Study participants

From November 2013 to March 2021, 187 patients with suspected cardiac tumors underwent CMR examinations. After applying the exclusion criteria, the cohort consisted of 80 patients, including 54 with benign cardiac tumors and 26 with primary malignant cardiac tumors.

Among patients with benign tumors, the most common subtype was myxoma (28/54, 52%), followed by rhabdomyoma (6/54, 11%) and fibroma (3/54, 5.6%). Among patients with primary malignant cardiac tumors, 18 had sarcoma (69%), 2 had lymphoma (7.7%), and 6 had unclassified malignant tumors (23%). Pathologically confirmed diagnoses were available for 37/54 and 20/26 patients with benign and primary malignant cardiac tumors, respectively. Of the 23 cases without pathological diagnoses (six rhabdomyomas, one fibroma, ten unclassified benign tumors, six unclassified primary malignant tumors), there was clear supporting evidence for the respective diagnoses. Detailed information about these 23 cases without pathological diagnosis is shown in Additional file 1: Table S2.

Demographic and clinical characteristics are presented in Table 1. Compared to the patients with primary malignant tumors, patients with benign tumors were less frequently male and had significantly higher blood pressure, higher level of hematocrit, and lower CA-125 levels.

**Table 1:** Demographic, clinical, and CMR characteristics in all subjects

Parameters	Benign cardiac tumors (n = 54)	Primary cardiac malignant tumors (n = 26)	Control subjects (n = 50)
Age, years	52 (34–65)	48 (30–57)	51 (42–60)
Males, n (%)	22 (41)^‡^	11 (42)*	24 (48)
BMI, kg/m^2^	22.9 ± 5.8	21.5 ± 4.8	22.4 (20.8–25.2)
SBP, mmHg	126 ± 17.3	108 ± 11.8*	122.4 ± 10.3
DBP, mmHg	81.4 ± 11.1	70.5 ± 10.4*	74.3 ± 7.8
Heart rate	83.0 ± 15.5^‡^	96.5 ± 17.9*^‡^	75.3 ± 9.6
Hct	0.39 ± 0.07^‡^	0.35 ± 0.08*^‡^	0.43 ± 0.03
cTnT, g/L	12.2 (7.0–45.7)	12.1 (9.8–22.2)	–
NT-proBNP, pg/mL	245 (57–871)	515 (295–1954)	–
AFP	2.2 (1.7–3.0)	2.4 (1.8–3.7)	–
CEA	1.7 (0.7–2.7)	1.4 (0.7–3.2)	–
CA19-9	9.9 (3.7–17.7)	7.4 (5.4–12.8)	–
CA125	21.0 (11.0–45.1)	150 (32.5–457)*	–
Cardiac structure and function			
LVEDVi, mL/m^2^	75.3 ± 20.2	68.4 ± 25.4	76.6 ± 12.2
LVESVi, mL/m^2^	30.5 ± 12.1	28.0 ± 10.8	28.5 ± 7.4
LVEF, %	59.7 ± 9.2	59.0 ± 7.6	63.4 ± 5.3
LVmassi, g/m^2^	63.4 ± 26.1	60.5 ± 30.4	45.5 ± 8.2
RVEDVi, mL/m^2^	67.2 ± 18.6	66.7 ± 36.4	70.7 ± 16.5
RVESVi, mL/m^2^	35.6 ± 17.7	33.3 ± 21.7	31.7 ± 10.3
RVEF, %	53.9 ± 11.0	53.6 ± 10.6	57.2 ± 14.2
Characteristics of tumors			
Location			
LA, n (%)	14 (26)	4 (15)	–
RA, n (%)	16 (30)	12 (46)	–
LV, n (%)	11 (20)	2 (8)	–
RV, n (%)	11 (20)	7 (27)	–
Valves, n (%)	2 (4)	1 (4)	–
Mobility, n (%)	37 (69)	4 (15)*	–
Invasiveness, n (%)	3 (6)	20 (77)*	–
Irregular border, n (%)	10 (19)	16 (62)*	–
Longest diameter, cm	3.3 (1.9–4.6)	6.5 (4.4–7.4)*	–
Shortest diameter, cm	2.0 (1.3–3.0)	3.2 (2.4–4.9)*	–
Pericardial effusion, n (%)	6 (11)	13 (54)*	–
Tissue characteristics			
T1-weighted, n (%)			
Hypointense	3 (6)	2 (3)	–
Isointense	48 (89)	21 (81)	–
Hyperintense	3 (6)	3 (12)	–
T2-weighted, n (%)			
Hypointense	3 (6)	2 (3)	–
Isointense	13 (24)	9 (35)	–
Hyperintense	38 (70)	15 (58)	–
First-pass perfusion, n (%)			
None	2 (4)	0 (0)	–
Hypoperfusion	32 (59)	13 (50)	–
Isoperfusion	8(15)	7 (27)	–
Hyperperfusion	12 (22)	6 (23)	–
LGE			
None	14 (26)	3 (12)	–
Homogeneous	13 (24)	3 (12)	–
Heterogeneous	27 (50)	20 (77)	–
Mapping parameters			
T1 mapping (pre)-mass, ms^a^	1684 ± 450	1627 ± 410	–
T1 mapping (post)-mass, ms^a^	433 ± 123	412 ± 135	–
T2 mapping-mass, ms^a^	59 ± 25	63 ± 34	–
Tissue characteristics of myocardium			
T1 mapping (pre-contrast)			
T1 mapping-mean, ms	1260 ± 46^‡^	1360 ± 61*^‡^	1206 ± 44
T1 mapping-peri, ms	1266 ± 47	1388 ± 96*	–
T1 mapping-remote, ms	1253 ± 51	1337 ± 67*	–
T1 mapping (post-contrast)			
T1 mapping-mean, ms	536 ± 63^‡^	544 ± 83^‡^	503 ± 52
T1 mapping-peri, ms	546 ± 66	540 ± 82	–
T1 mapping-remote, ms	544 ± 63	577 ± 75	–
T2 mapping			
T2 mapping-mean, ms	41 ± 3.0^‡^	42 ± 3.2^‡^	38 ± 3.1
T2 mapping-peri, ms	40 ± 3.1	42 ± 3.2	–
T2 mapping-remote, ms	41 ± 3.0	41 ± 3.7	–
ECV			
ECV-mean, %	30 ± 2.5^‡^	35 ± 5.2*^‡^	27 ± 3.0
ECV-peri, %	29 ± 3.0	36 ± 6.9*	–
ECV-remote, %	29 ± 2.7	33 ± 5.8*	–
LGE			
Presence, n (%)	0	3 (12%)	–

Abnormally distributed variables were presented as median with interquartile range and compared using the Mann–Whitney U test

*CMR* cardiovascular magnetic resonance, *BMI* body mass index, *SBP* systolic blood pressure, *DBP* diastolic blood pressure, *Hct* haematocrit, *cTNT* cardiac troponin T, *NT-proBNP* N-terminal pro-B-type natriuretic peptide, *AFP *alpha-fetoprotein, *CEA* carcinoembryonic antigen, *CA19-9* cancer antigen 19-9, *CA125* cancer antigen 125, *LVEDVi* left ventricular end-diastolic volume index, *LVESVi* left ventricular end-systolic volume index, *LVEF* left ventricular ejection fraction, *LVmassi* left ventricular mass index, *RVEDVi* right ventricular end-diastolic volume index, *RVESVi* right ventricular end-systolic volume index, *RVEF* right ventricular ejection fraction, *LA* left atrium, *RA* right atrium, *LV* left ventricle, *RV* right ventricle

*P value indicates significant difference between benign and primary malignant cardiac tumors

^‡^P value indicates significant difference in the mean myocardial values compared with normal controls

^a^The data are obtained from patients with mapping values on cardiac mass

### CMR analyses

We performed the comparison of cardiac function and volumes among the two groups of patients with cardiac tumors and normal controls. There were no significant differences in LV volume index, LV mass index, LVEF, RV volume index, or RVEF among the groups (Table 1). In patients with primary malignant tumors, 3 patients showed positive myocardial LGE. One of the two patients diagnosed with undifferentiated sarcoma showed subepicardial LGE in the LV free wall, the other showed septal mid myocardial linear LGE. The 3rd patient was diagnosed with liposarcoma and exhibited patchy LGE in the middle level of LV. Meanwhile, no patients with benign tumors or normal controls had myocardial LGE.

### Characteristics of cardiac tumors

Traditional morphological and tissue characteristics of cardiac tumors are presented in Table 1. Significant differences were found in characteristics such as tumor mobility, invasiveness, irregularity of border, size, and pericardial effusion when comparing benign and malignant tumors. On the other hand, the traditional tissue characteristics of tumors such as T1W, T2W, first pass perfusion, and LGE heterogeneity did not show significant differences between the groups.

### Parametric mapping of cardiac tumors

**Fig. 3:**
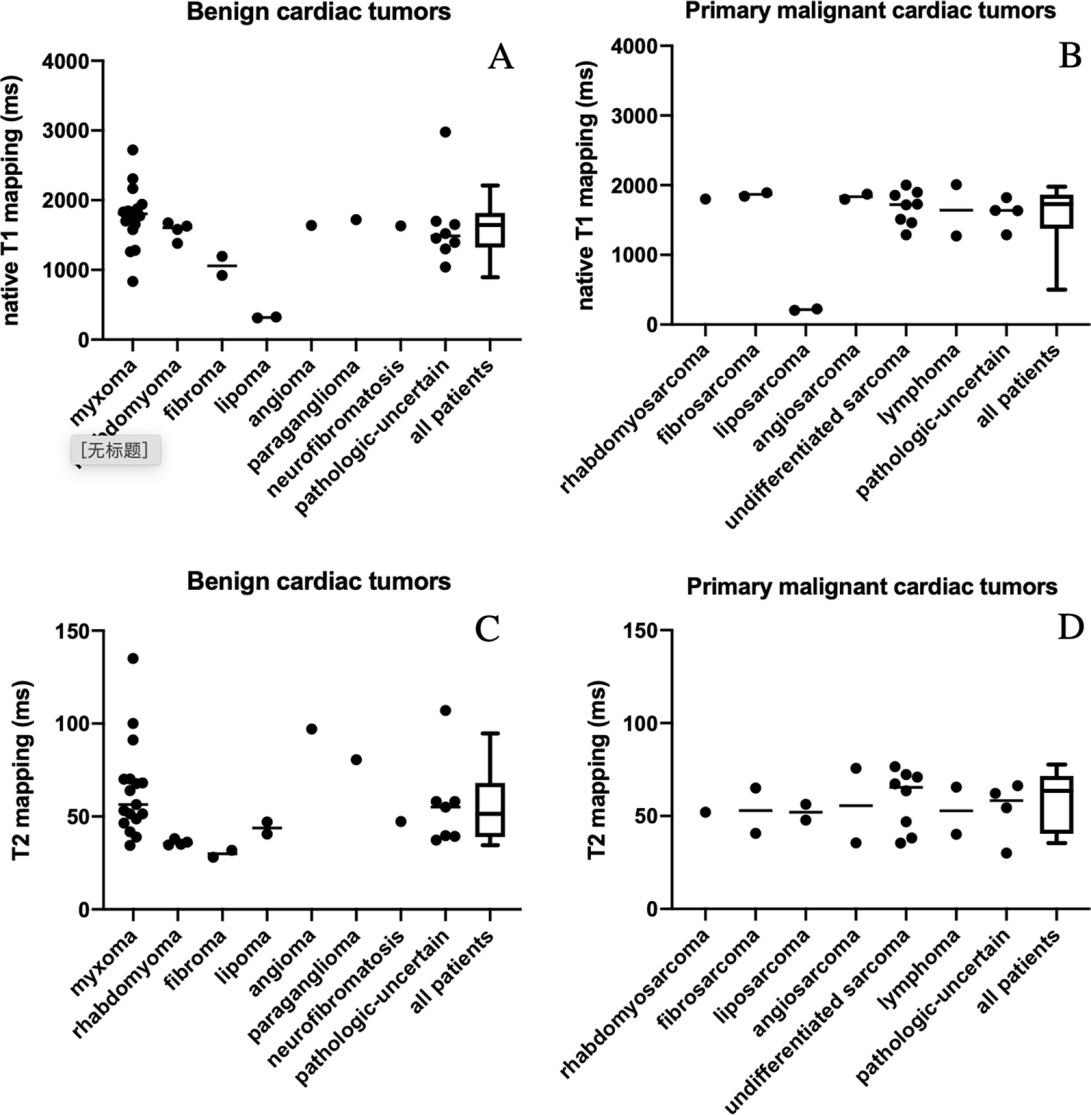
Native T1, T2 values of cardiac tumors in diverse pathological groups. **A** Native T1 mapping values of the cardiac tumors in patients with benign cardiac tumors and **B** primary malignant cardiac tumors; **C** T2 mapping values of the cardiac tumors in patients with benign cardiac tumors and **D** primary malignant cardiac tumors

We measured the T1 and T2 relaxation values of the cardiac tumors in the primary malignant group (T1 value: 1627 ± 410 ms, T2 value: 63 ± 34 ms) and benign group (T1: 1684 ± 450 ms, T2: 59 ± 25 ms). No significant intergroup differences were found due to the large tissue heterogeneity within the groups. We measured the T1 and T2 values of tumors with different pathologic types to explore whether the T1 and T2 mapping could suggest different tissue origin (Table 2). Myxomas showed T1 and T2 values of 1744 ± 434 ms and 64 ± 25 ms, respectively, due to their high water content. In the two patients with lipoma, the T1 and T2 relaxation values were 312 and 47 ms, and 315 and 46 ms, respectively, while the two liposarcomas showed T1 of 205 and 56 ms, and 225 ms and 48 ms, respectively. In both groups, the T1 values were very low, indicating that T1 may have some value in distinguishing fatty tissue. Furthermore, we compared benign and malignant tumors of the same histological type in a few cases. The two patients with fibroma showed T1 and T2 values of 1194 and 32 ms, and 921 and 28 ms, respectively, while the two fibrosarcoma patients showed 1893 and 65 ms, and 1843 and 45 ms, respectively. The T1 and T2 values of angioma tumor (n = 1) were 1637 ms and 87 ms, respectively, and the T1 and T2 values were 1798 ms and 76 ms, and 1872 and 36 ms in the angiosarcoma patients (n = 2). The T1 and T2 values of the paraganglioma group, which are also rich in blood signals, were respectively 1720 and 81 ms. The T1 and T2 values of the fibroma group (n = 2) were 921 and 28 ms, and 1194 and 32 ms, while the T1 and T2 values of the fibrosarcoma group (n = 2) were 1893 and 65 ms, and 1843 and 41 ms, respectively. The distribution of T1 and T2 values of each histological subtype of cardiac tumor was presented in Fig. 3.

**Table 2:** Diagnostic performance of T1, T2 mapping, and ECV parameters of myocardium in discriminating between primary malignant cardiac tumors and benign cardiac tumors

	AUC	Cutoff value	Sensitivity (%)	Specificity (%)
Native T1 mapping (ms) of myocardium				
Mean	0.919	1300	89	87
Peri-	0.889	1314	85	85
Remote-	0.832	1308	65	89
T2 mapping (ms) of myocardium				
Mean	0.619	40.8	73	54
Peri-	0.660	40.6	49	63
Remote-	0.544	41.3	50	65
ECV (%) of myocardium				
Mean	0.817	31.3	85	70
Peri-	0.801	32.1	73	85
Remote-	0.750	31.7	69	83

### T1 mapping and ECV measurements of the myocardium

**Fig. 4:**
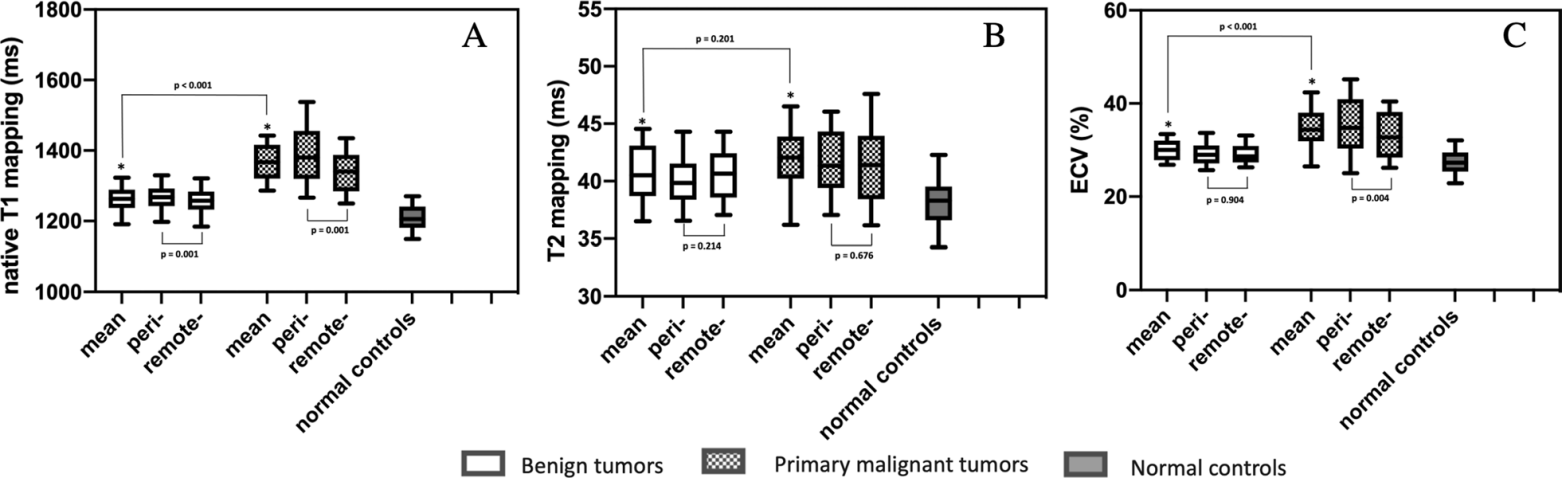
Comparison of mean, peri-, and remote myocardial native T1, T2, and ECV values in patients with benign and primary malignant cardiac tumors. Comparison of mean, peri-, and remote myocardial (**A**) native T1 values in patients with benign and primary malignant cardiac tumors, **B** T2 values in patients with benign and primary malignant cardiac tumors, **C** ECV values in patients with benign and primary malignant cardiac tumors. The lower and upper limits of the box represent the 25th and 75th percentiles and whiskers represent the 10th to 90th percentile range. * Above the boxplot indicates significant difference between the group of patients and normal controls

**Fig. 5:**
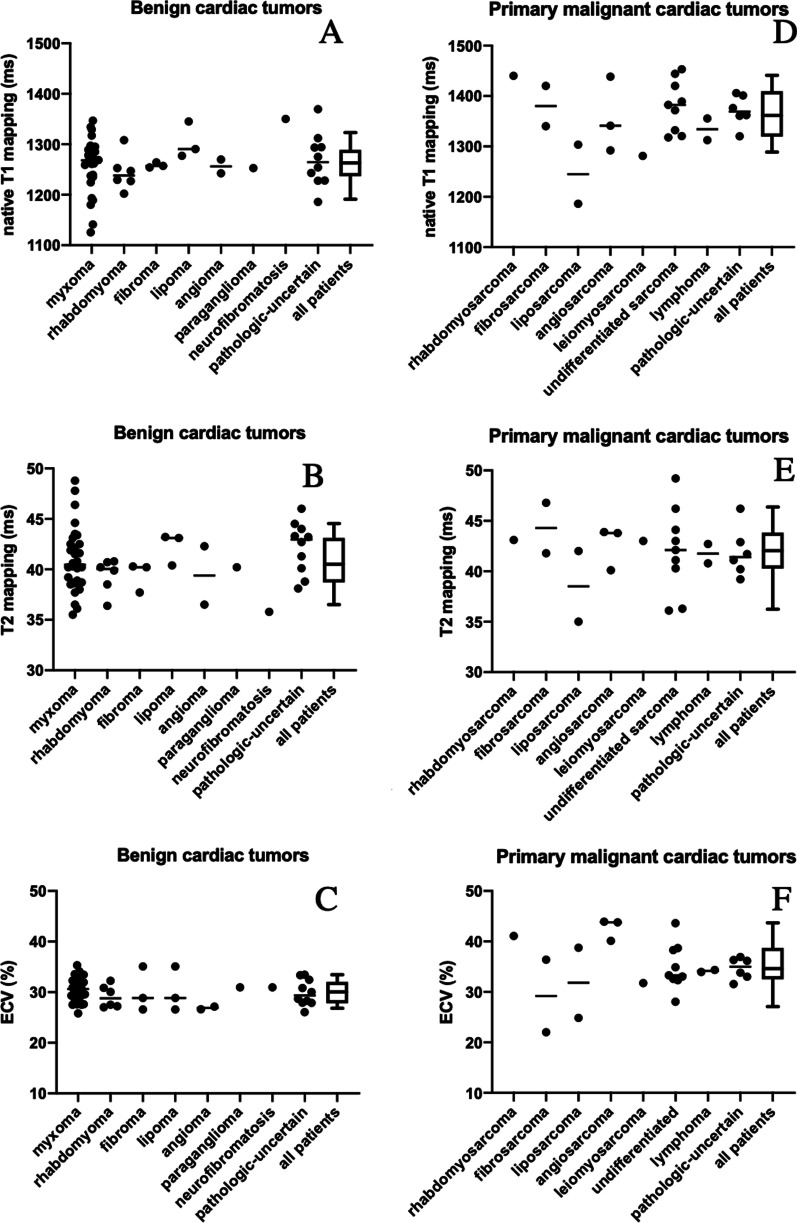
T1, T2, and ECV values of myocardium in diverse pathological groups. **A** Native T1 mapping values of the cardiac tumors in patients with benign cardiac tumors, **B** primary malignant cardiac tumors, **C** T2 mapping values of the cardiac tumors in patients with benign cardiac tumors, **D** primary malignant cardiac tumors, and **E** ECV values of the cardiac tumors in patients with benign cardiac tumors, **F** primary malignant cardiac tumors

Patients with primary malignant cardiac tumors showed significantly higher mean myocardial T1 values than those with benign cardiac tumors (1360 ± 61 ms vs. 1260 ± 46 ms, *P* < 0.001). Both groups of patients showed significantly higher levels of mean T1 values compared with normal controls (1206 ± 44 ms, *P* < 0.001) (Table 1 and Fig. 4A). The AUC of mean T1 value to differentiate primary malignant and benign tumors was 0.917 (cutoff value, 1300 ms; sensitivity, 89%; specificity, 87%) (Table 3). Further, we compared the myocardial T1 values in adjacent and remote areas. In patients with primary malignant cardiac tumors, the peri-tumor area showed significantly higher myocardial T1 value than remote-tumor area (1388 ± 96 ms vs. 1337 ± 67 ms, *P* < 0.001), there was no significant difference in patients with benign tumors (1266 ± 47 ms vs. 1253 ± 51 ms, *P* < 0.001) (Table 1 and Fig. 4A). The myocardial T1 in patients with different pathologic types are shown in Fig. 5.

**Table 3:** The LV myocardial native T1, T2 mapping, and ECV valued in benign and primary malignant cardiac tumors in different locations

	LA	RA	LV	RV	Valves
Benign					
T1 mapping-mean (ms)	1287 ± 54	1269 ± 40	1239 ± 35	1254 ± 26	1197 ± 101
T2 mapping-mean (ms)	41 ± 4.0	42 ± 2.8	40 ± 2.2	40 ± 3.5	39 ± 4.3
ECV-mean	30 ± 2.3	32 ± 5.4	30 ± 2.8	29 ± 2.4	30 ± 4.0
Primary malignant					
T1 mapping-mean (ms)	1330 ± 14*	1379 ± 50*	1357 ± 36*	1376 ± 69*	1186
T2 mapping-mean (ms)	43 ± 5.4	41 ± 2.2	40 ± 4.7	44 ± 3.2	35
ECV-mean (%)	35 ± 2.3*	34 ± 5.1*	38 ± 7.5*	37 ± 6.0*	25

*P value indicates significant difference between benign and primary malignant cardiac tumors at the corresponding location

For the evaluation of myocardial ECV, patients with primary malignant tumors showed significantly higher ECV values than those with benign cardiac tumors (35% ± 5.2% vs. 30% ± 2.5%, *P* < 0.001), and both groups showed significantly higher level than normal controls (27% ± 3.0%, *P* < 0.001) (Table 1 and Fig. 4C). The AUC of mean ECV to differentiate primary malignant and benign tumors was 0.817 (cutoff value, 31%; sensitivity, 85%; specificity, 70%) (Table 3). In the primary malignant group, the peri-tumor ECV was significantly higher than the remote-tumor ECV (36% ± 6.9% vs. 33% ± 5.8%, *P* < 0.001). However, this trend was not found in the benign group (Fig. 4C). The myocardial ECV in patients with different pathologic types are shown in Fig. 5.

### T2 mapping measurements of the myocardium

Patients with both primary malignant and benign cardiac tumors showed a significantly higher level of mean T2 values than normal controls (42 ± 3.2 ms, 41 ± 3.0 ms vs. 38 ± 3.1 ms, *P* < 0.001). However, there was no significant difference in mean T2 values between patients with primary malignant cardiac tumors and patients with benign cardiac tumors (42 ± 3.2 ms vs. 41 ± 3.0 ms). In addition, no significant difference was observed in peri- and remote-tumor myocardial T2 values in either malignant or benign groups (Table 1, Fig. 4B). The AUC of mean T2 value to differentiate primary malignant and benign tumors was only 0.619 (Table 3). The myocardial T2 values in patients with different pathologic types are shown in Fig. 5.

### The potential effect of cardiac tumor location on the surrounding myocardium

The location of cardiac tumors were classified based on the chamber localization (right atrium [RA], right ventricle [RV], left atrium [LA], LV), or valve involvement. The results showed that when compared with benign cardiac masses, primary malignant cardiac tumors were more commonly located in the RA and less commonly located in the LV (Table 1). Further analysis of the effect of the tumor location on myocardial tissue characteristics showed that in patients with tumor located in LV, RV, LA, and RA, respectively, myocardial T1 mapping and ECV values of the malignant tumor group were significantly higher than those of the benign group (Table 4). In addition, we subgrouped the location of tumors by intra-cavitary (predominantly localized to the cardiac chamber) and intramural (invading into the myocardium). The mean native T1 value of the myocardium surrounding the tumor in the intramyocardial group is significantly higher than that of the intra-cavitary group (Table 4). The summary of the size and mapping values in patients with different pathology and tumors locations are presented in Additional file 1: Table S1.

**Table 4:** LV myocardial native T1, T2 mapping, and ECV valued in cardiac tumors localized in intra-cavitary or intramural

	Intra-cavitary	Intramural	P value
T1 mapping-mean (ms)	1280 ± 66	1313 ± 73.5	0.055
T1 mapping-peri (ms)	1283 ± 78	1338 ± 100	**0.014**
T1 mapping-remote (ms)	1267 ± 70	1301 ± 66.6	0.053
T2 mapping-mean (ms)	41 ± 3.2	42 ± 3.2	0.595
T2 mapping-peri (ms)	40 ± 3.3	41 ± 3.5	0.275
T2 mapping-remote (ms)	41 ± 3.4	42 ± 3.7	0.120
ECV-mean (%)	31 ± 2.8	33 ± 6.5	**0.029**
ECV-peri (%)	30 ± 4.0	33 ± 7.6	**0.026**
ECV-remote (%)	30 ± 3.2	32 ± 6.3	0.101

*LV* left ventricle, *ECV* extracellular volume fraction

### Intra- and interobserver reproducibility

We randomly selected 30 healthy participants and 30 patients with cardiac tumors to performed intra-observer and inter-observer analyses of T1, T2, and ECV values. The results of the Bland–Altman analysis, CoV, and ICC are presented in Additional file 1: Table S3.

## Discussion

In this study, we utilized CMR T1, T2 mapping techniques to explore the tissue characteristics of cardiac tumors and myocardium among patients with benign and primary malignant cardiac tumors. The main findings of our study are (a) patients with primary cardiac tumors showed significantly higher myocardial T1 and ECV values compared to those with benign tumors; (b) myocardial T1 mapping showed good diagnostic value in differentiating between benign and primary malignant tumors; and (c) it is difficult to distinguish benign and malignant tumors based on tissue mapping of cardiac tumors themselves due to the high heterogeneity of the tissue origin of the tumors.

To our knowledge, this is the first study to comprehensively investigate quantitative tissue characteristics of cardiac tumors and myocardium by using multi-parametric mapping techniques of CMR. In the current clinical applications of non-invasive imaging, preliminary assessment of the nature of cardiac tumors is mainly made based on the morphologic features, location, invasiveness, and perfusion characteristics. However, distinguishing malignant from benign tumors using these characteristics can be difficult when tumors lack typical characteristics, such as invasiveness. Shenoy et al*.* reported that CMR tissue characterization include T1- and T2-weighted imaging provided high accuracy for differentiation of benign and malignant lesions [10]. In another study, Nasser et al*.* indicated the potential value of CMR mapping techniques for differentiating cardiac myxomas from other cardiac tumor entities with myxomas having elevated native T1, T2, and ECV values in comparison with normal myocardium [31].

Given the advantage of CMR T1 and T2 mapping techniques to visualize and quantify histological composition [20], we explored the value of quantitative parameters in differentiating benign and malignant tumors. We found that due to the large heterogeneity among benign and malignant tumors, no significant between-group differences were observed. However, several typical characteristics in different pathological types of tumors were observed. For example, patients with lipoma and liposarcoma both showed significantly decreased T1 values compared to the myocardium, which may indicate the presence of mature fat [32]. We found that the heterogeneity of the mapping values in several pathological tumor types is high, which maybe related to the different tissue origins, and is not specific to the benign or malignant nature of the tissue. Pathological changes such as necrosis and hemorrhage may also occur in the tumors, which will affect the mapping values. In addition, we found that some cardiac tumors could be barely recognized in mapping images due to their small size or high mobility, thus mapping data could not be obtained confidently in these cases. Future larger studies focusing on the tissue characteristics of cardiac tumors may provide additional diagnostic value.

We found that myocardial LGE and mapping techniques may have complementary value in delineating the effect of cardiac tumors on the myocardium. While patients with primary cardiac diseases such as myocardial ischemia or cardiomyopathy, often present with myocardial LGE, patients with cardiac tumors present with limited myocardial LGE (0 cases of benign tumors, 3 cases of malignant tumors). In the three patients with malignancy, myocardial LGE may be caused by the invasion of tumor into the myocardium. Thus, the presence and pattern of myocardial LGE may help us determine whether patients have primary myocardial damage.

For the analyses of myocardial mapping values, we found that patients with primary malignant cardiac tumors showed significantly higher myocardial T1 and ECV values compared to patients with benign tumors, and myocardial T1 cutoff value of 1300 ms showed good diagnostic value (AUC = 0.919). Beroukhim et al*.* found that imaging sequences currently available by CMR could not distinguish between benign hemangioma and malignant angiosarcoma as well as tumors with sufficient vascular supply such as paraganglioma [33]. In our study, although the sample size was limited, we found the myocardium T1 value of the malignant angiosarcoma (N = 3, 1438 ms, 1341 ms, 1292 ms) to be significantly higher compared to myocardial T1 in patients (N = 2) with hemangioma (T1 = 1270 ms, and 1242 ms) and paraganglioma (N = 1, 1253 ms), suggesting that the myocardium T1 value may have clinical significance in differentiating benign and malignant vascular tumors. In addition, the T1 value of the peri-tumor myocardium is significantly higher than the T1 value of remote myocardium. This difference is most obvious in the primary malignant cardiac tumors group, and may be caused by tumor infiltration and activation of surrounding fibroblasts.

The potential effects of cardiac tumors on cardiac function, structure, and volumes are of great clinical significance. Previous studies reported that cardiac tumors could cause acute heart failure and sudden death [34–37]. Primary or secondary cardiac malignancies causing peripheral or distant myocardial necrosis have also been reported [32, 38]. Microscopically, the infiltration of malignant tumors into surrounding tissues and the damage caused by tumors will lead to the activation of fibroblasts, and long-term stimulation may cause increased levels of fibrosis in the surrounding tissues and even the entire heart, as well as microvascular dysfunction [18]. In contrast, with the slow growth and complete fibrous capsule of benign tumors, the effects of benign tumors on the surrounding myocardium are limited. The slight increase of the T1 value of myocardium in benign tumors may be related to the compression of the surrounding tissues of tumors. It is curious as to why an RA lesion would affect LV myocardial tissue properties, we speculate that this may be related to the presence of tumor paracrine factors [39] or microscopic tumor infiltration [32, 38]. Future studies are needed to identify the potential mechanisms that induce pathophysiological changes of the myocardium since we could not decipher the mechanism of remote “mass effect” on the LV myocardium from this study.

Not all malignant tumors can be correctly diagnosed by CMR. A patient with dedifferentiated liposarcoma was initially misdiagnosed as myxoma due to its regular borders and the lack of infiltration or invasive features. The average T1 value of the surrounding myocardium was 1186 ms, showing no significant increase. Postoperative pathology confirmed an dedifferentiated liposarcoma, indicating the importance of pathological examination as the gold standard, and the location of the tumor (for example, in the heart cavity), and its size. The case was previously reported [40].

This study has several limitations. First, the sample size is relatively small, we did not perform the comparison based on different locations among patients with the same type of pathology in the current study. Second, not all patients underwent pathological examination. In clinical practice, some benign tumors, such as rhabdomyoma, fibromas, and fibroelastoma, are usually discovered incidentally. For asymptomatic patients with relatively small-sized masses and typical images characteristic of benign tumors, surgical intervention was not recommended [41, 42]. Thus, the study includes a group of patients with typical clinical and imaging characteristics and without surgical indications. Some patients with a high degree of suspicion of the malignant tumor did not undergo surgery either due to high surgical risk or the patient declining therapy. We did not exclude these patients to avoid introducing additional bias.

In summary, while it is difficult to distinguish benign and malignant tumors based on mapping values of cardiac tumors due to high heterogeneity, our results indicate that patients with malignant cardiac tumors have higher T1 and ECV values than those with benign cardiac tumors, which highlights the potential value of myocardial T1 mapping in differentiating between primary malignant and benign cardiac tumors. Due to the rarity of cardiac tumors, a large multi-center CMR study using T1, T2 mapping techniques are needed in the future to explore cardiac tumors with different pathological types and their effects on the myocardium.

## Conclusion

In conclusion, this is the first study to comprehensively investigate the quantitative tissue characteristics of cardiac tumors and myocardium by using multi-parametric mapping techniques of CMR. While it was difficult to distinguish benign and malignant cardiac tumors based on mapping of cardiac tumors due to the high heterogeneity, we found that patients with primary cardiac tumors showed significantly higher myocardial T1 and ECV values than those with benign tumors. Myocardial T1 mapping showed good diagnostic value between benign and primary malignant tumors, which may serve as a new imaging marker for primary malignant cardiac tumors.

## Supplementary Information

**Additional file 1. **Supplementary Methods and Materials. **Table S2.** Supplementary Table 2.

## Data Availability

The datasets acquired and/or analyzed during the current study are available from the corresponding author on reasonable request.

## Abbreviations

AFP: Carcinoembryonic antigen
AUC: Area under the curve
BSA: Body surface area
CA19-9: Cancer antigen 19-9
CEA: Cancer antigen 19-9
CK: Creatine kinase
CK-MB: Creatine kinase-myocardial band
CMR: Cardiovascular magnetic resonance
CoVs: Coefficients of variation
CRP: C-reaction protein
cTnT: Cardiac troponins T
DBP: Diastolic blood pressure
ECV: Extracellular volume
FOV: Field of view
Hct: Haematocrit
HF: Heart failure
ICCs: Intra-class correlation coefficients
IQR: Interquartile range
LA: Left atrium
LGE: Late gadolinium enhancement
LVEF: Left ventricular ejection fraction
LVEDVi: Left ventricular end-diastolic volume index
LVESVi: Left ventricular end-systolic volume index
LVmassi: Left ventricular mass index
MOLLI: Modified Look-Locker inversion recovery
NT-proBNP: N-terminal pro b-type natriuretic peptide
PET-CT: Positron emission tomography-computed tomography
RA: Right atrium
ROC: Receiver operator characteristic
ROI: Region of interest
RVEF: Right ventricular ejection fraction
RVEDVi: Right ventricular end-diastolic volume index
RVESVi: Right ventricular end-systolic volume index
SCMR: Society of Cardiovascular Magnetic Resonance
SD: Standard derivation
SI: Signal intensity
SSFP: Steady-state free precession
STIR: Short-tau inversion recovery
T2W: T2-wighted ratio
TE: Echo time
TR: Repetition time
