# Supplementary material for: Multiparametric mapping by cardiovascular magnetic resonance imaging in cardiac tumors
Source: J Cardiovasc Magn Reson. 2023 Jun 22;25:37. doi: 10.1186/s12968-023-00938-9 (PMC10286406; doi:10.1186/s12968-023-00938-9)
Supplement: Supplementary file 1 — Additional file 1. Supplementary Methods and Materials. Table S2. Supplementary Table 2. [file 12968_2023_938_MOESM1_ESM.docx]

**Additional Methods and Materials**

Typical scan parameters

The typical scan parameters were as follows: ﻿Steady-state free precession (SSFP) cine images were acquired with repetition time (TR), 2.6 ms; echo time (TE), 1.3 ms; flip angle, 50°; field of view (FOV), 280 × 340 mm2; matrix size, 162 × 192; average temporal resolution, 35–45 ms; and slice thickness, 8 mm with no gap. T1 mapping images were acquired by using the Modified Look-Locker Inversion- recovery (MOLLI) sequence with a 5b(3b)3b (b stands for heartbeat) scheme and a 4b(1b)3b(1b)2b scheme before and 10–15 min after the gadolinium injection on three short-axis and 4-chamber views. The typical scan parameters were as follows: TR, 300 ms; TE, 1.2 ms; flip angle, 35°; FOV, 270 × 320 mm2; matrix size, 144 × 256; and slice thickness, 8 mm. T2 mapping images were acquired by T2-prepared single-shot bSSFP technique before administration of gadolinium on the short- and long-axis views identical to those associated with T1 mapping images. The typical scan parameters were as follows: TR, 240 ms; TE, 1.0 ms; flip angle, 12°; FOV, 320 × 340 mm2; matrix, 114 × 176; slice thickness, 8 mm; and T2 preparation pulse with 0, 30, 55 ms spin echo times.

**Subject overlap with previously published works**

In the study, we included patients diagnosed with cardiac tumors who underwent cardiac MRI between November 2011 and March 2021. In a previous study, we reported on 76 patients included in the current study. The prior developed a quantitative score algorithm for differentiating benign and malignant cardiac tumors based on conventional CMR parameters including invasiveness, irregular border, heterogeneous LGE of tumors ^1^. The current study updates the number of patients with this continuously prospective study and includes multi-parametric mapping values of cardiac tumors and myocardium into analyses.

In addition, the 50 age- and sex-matched normal controls in the study were selected from a CMR database of healthy Chinese populations established by our center. Studies of normal reference of this group of the normal controls have been published before ^2,3^.

**The detailed information of these 23 cases without pathological diagnosis**

Six cases were diagnosed with rhabdomyoma based on CMR imaging characteristics (they appeared isointense to normal myocardium on T1-weighted images and hyperintense on T2-weighted images, and showed no enhancement with gadolinium-based contrast). The patients were all children and asymptomatic. Considering that rhabdomyoma could regress spontaneously, these patients did not receive surgical intervention. One case was fibroma with typical CMR features (isointense relative to normal myocardium on T1-weighted images, characteristically hypointense on T2-weighted images, and hyperenhancement on LGE images). Ten cases were considered as unclassified benign tumors based on CMR imaging because the tumor borders were clear without infiltration of surrounding tissues, the systemic symptoms were mild, there was no extracardiac involvement, and no large amount of pericardial effusion. Among them, three cases underwent PET-CT, and the tumors showed low uptake of 18F-Fluorodeoxyglucose (FDG). Because most of these patients were asymptomatic and refused surgery or biopsy, no pathological diagnosis results were available. However, according to the mild symptoms, imaging features, and long-term survival without intervention, these patients were classified as unclassified benign tumor subgroup and included in the study. Six cases were considered as primary cardiac malignancies because of their imaging characteristics, large amounts of pericardial effusion, elevated tumor markers, and poor survival. CMR showed infiltration of surrounding tissues and irregular boundaries, and PET-CT showed significantly increased 18F-FDG uptake at tumor sites. These patients refused surgery or cardiac biopsy and died soon after, so they were categorized into the malignant tumor group as unclassified malignant tumors.

Table S1. The summary of the size and mapping values in patients with different diagnoses and mass location.

| Diagnosis | Location | No. | Longest diameters | Shortest diameters | T1 mapping (mass) | T2 mapping (mass) | T1 mapping (myo) | T2 mapping (myo) | ECV (myo) |  |
| --- | --- | --- | --- | --- | --- | --- | --- | --- | --- | --- |
| **Benign** |  |  |  |  |  |  |  |  |  |  |
| Myxoma |  | 28 |  |  |  |  |  |  |  |  |
|  | LA | 11 | 4.0(2.0-5.0) | 2.2(1.7-3.7) | 1803(1171-1894) | 48(40-56) | 1273.1 ± 53.8 | 40.4 ± 3.6 | 31.0 ± 1.8 |  |
|  | RA | 9 | 4.2(2.5-5.6) | 2.9(2.2-4.2) | 1832(1518-2813) | 64(46-113) | 1251.5 ± 49.9 | 42.0 ± 2.6 | 30.3 ± 2.9 |  |
|  | LV | 2 | 2.1/1.8 | 1.5/1.2 | 1728/1546 | 100/82 | 1193.0/1224.6 | 42.1/43.1 | 29.5/30.9 |  |
|  |  |  |  |  |  |  |  |  |  |  |
|  | RV | 4 | 3.3(1.6-4.8) | 1.7(1.1-2.4) | 1678(1598-2052) | 69(59-70) | 1277.0 ± 8.9 | 41.5 ± 4.3 | 30.2 ± 3.1 |  |
|  | Valves | 2 | 7.0/2.1 | 5.1/1.4 | 1843 | 51.4 | 1125.6/1269.0 | 35.5/ 41.6 | 27.5/33.1 |  |
| Rhabdomyoma | | 6 |  |  |  |  |  |  |  |  |
|  | LV | 2 | 2.1/1.7 | 0.9/0.8 | 1383 | 35 | 1229.5/1252.7 | 40.2/40.8 | 27.0/30.0 |  |
|  | RV | 4 | 1.7(1.1-5.0) | 1.1(0.8-1.7) | 1629(1582-1703) | 36(35-37) | 1246 ± 45.2 | 38.9 ± 1.9 | 29.4 ± 2.5 |  |
| Fibroma |  | 3 |  |  |  |  |  |  |  |  |
|  | LV | 2 | 6.5/3.7 | 4.2/2.4 | 1194/921 | 31.8/28.0 | 1254.0/1264.0 | 40.2/40.3 | 26.6/35.1 |  |
|  | RV | 1 | 1.8 | 1.5 | 1203 | 34 | 1257 | 37.7 | 28.9 |  |
| Lipoma |  | 3 |  |  |  |  |  |  |  |  |
|  | LA | 1 | 2 | 0.8 | - | - | 1345.2 | 43.2 | 31.1 |  |
|  | RA | 1 | 4.3 | 2.7 | 312 | 47 | 1277.3 | 43.1 | 25.7 |  |
|  | LV | 1 | 1.6 | 1 | 325 | 41 | 1290.6 | 40.4 | 29.5 |  |
| Angioma |  | 2 |  |  |  |  |  |  |  |  |
|  | LV | 1 | 1.9 | 1.1 | 1637 | 87 | 1269.9 | 36.5 | 27.2 |  |
|  | RV | 1 | 2.1 | 1.9 | - | - | 1242.4 | 42.3 | 26.6 |  |
| Paraganglioma | | 1 |  |  |  |  |  |  |  |  |
|  | LA | 1 | 4 | 3.9 | 1720 | 81 | 1252.9 | 40.2 | 30.9 |  |
| Neurofibromatosis | | 1 |  |  |  |  |  |  |  |  |
|  | RA | 1 | 2.4 | 2.3 | 1630 | 47 | 1350 | 35.8 | 31 |  |
| Unclassified | | 10 |  |  |  |  |  |  |  |  |
|  | LA | 2 | 5.3/4.4 | 2.6/2.2 | 1699 | 42 | 1293.5/1269.5 | 46.0/44.5 | 27.9/26.1 |  |
|  | RA | 3 | 3.8(2.8-5.0) | 2.2(1.5-3.4) | 1042/1302/1456 | 37/39/58 | 1274.4 ± 19.9 | 41.4 ± 2.6 | 31.4 ± 2.7 |  |
|  | LV | 4 | 1.8(1.5-2.0) | 1.3(1.2-1.4) | 1043/1302/1457 | 37/39/58 | 1221.1 ± 24.6 | 41.0 ± 2.4 | 30.0 ± 2.4 |  |
|  | RV | 1 | 3.2 | 2.4 | 2978 | 107 | 1262 | 43.3 | 30.8 |  |
| **Primary Malignant** | |  |  |  |  |  |  |  |  |  |
| Rhabdomyosarcoma | | 1 |  |  |  |  |  |  |  |  |
|  | RA | 1 | 7.1 | 5.6 | 2978 | 107 | 1440 | 43.1 | 41.1 |  |
| Fibrosarcoma | | 2 |  |  |  |  |  |  |  |  |
|  | LA | 1 | 6.4 | 3.2 | 1843 | 41 | 1340.3 | 46.8 | 36.4 |  |
|  | RA | 1 | 9.2 | 5.1 | 1893 | 65 | 1420.2 | 41.8 | 22 |  |
| Liposarcoma | | 2 |  |  |  |  |  |  |  |  |
|  | RV | 1 | 6.5 | 2.3 | 204 | 148 | 1303.4 | 42.1 | 38.8 |  |
|  | Valves | 1 | 3.2 | 2.4 | 215 | 78 | 1186.6 | 35 | 24.7 |  |
| Angiosarcoma | | 3 |  |  |  |  |  |  |  |  |
|  | RA | 2 | 4.7/6.9 | 2.5/3.0 | 1872 | 36 | 1292.7/1241.2 | 40.1/43.8 | 30.5/37.3 |  |
|  | RV | 1 | 7.4/3.5 | 43.9 | 1798 | 76 | 1438.4 | 43.9 | 46.6 |  |
| leiomyosarcoma | | 1 |  |  |  |  |  |  |  |  |
|  | RV | 1 | 5.4 | 1.2 | - | - | 1281 | 43 | 31.8 |  |
| Undifferentiated sarcoma | | 9 |  |  |  |  |  |  |  |  |
|  | RA | 4 | 5.8(3.9-7.2) | 3.6(2.7-4.9) | 1855/1900/2000 | 67/71/72 | 1377.9 ± 69.1 | 40.9 ± 3.4 | 34.5 ± 2.8 |  |
|  | LV | 2 | 22.0/4.2 | 13.0/2.6 | 1462/1728 | 35/47 | 1332.0/1382.6 | 36.3/43.0 | 33.0/43.6 |  |
|  | RV | 3 | 11.5/5.5/5.3 | 4.5/4.1/1.7 | 1287/1510/1717 | 38/64/77 | 1444.2/1389.0/1371.7 | 49.2/46.2/40.3 | 38.7/33.3/28.1 |  |
| Lymphoma | | 2 |  |  |  |  |  |  |  |  |
|  | RA | 2 | 6.4/3.3 | 4.6/1.4 | 1272/2009 | 40/66 | 1355.6/1312.4 | 42.7/40.8 | 34.4/33.9 |  |
| Unclassified | | 6 |  |  |  |  |  |  |  |  |
|  | LA | 2 | 7.5/7.2 | 5.0/2.8 | 1287 | 30 | 1376.5/1320.4 | 41.1/39.2 | 36.9/33.0 |  |
|  | RA | 3 | 7.7/6.5/4.4 | 6.3/4.9/3.1 | 1635/1639 | 62/66 | 1405.8/1362.0/1360.8 | 42.9/41.7/40.2 | 36.3/36.2/31.5 |  |
|  | RV | 1 | 9.5 | 3.5 | 1821 | 54 | 1401.2 | 46.2 | 33.8 |  |

^*^For groups with a sample size of less than 3, we directly present the value of each patient

Table S2. The detailed information in 23 cases without pathological diagnosis.

| Cases | Age  (year) | gender | Longest diameter  (cm) | location | Activity (no:0;  yes:1) | border  (regular:0;  irregular:1) | Invasiveness  (no:0;yes:1) | Delayed enhancement (no:0;homogeneous:1;  heterogeneous:2) | pericardial effusion  (no:0;yes:1) | survival time  (month) |
| --- | --- | --- | --- | --- | --- | --- | --- | --- | --- | --- |
| 1 | 6 | male | 1.69 | LV | 1 | 1 | 0 | 0 | 0 | Survival/69.9 |
| 2 | 9 | female | 1.32 | RV | 1 | 0 | 0 | 0 | 0 | Survival/63.8 |
| 3 | 3 | male | 0.92 | RV | 0 | 0 | 0 | 0 | 0 | Survival/57.2 |
| 4 | 4 | female | 2.1 | LV | 1 | 0 | 0 | 0 | 0 | Survival/49.9 |
| 5 | 3 | female | 1.5 | RV | 1 | 0 | 0 | 0 | 0 | Survival/48.8 |
| 6 | 12 | male | 6.08 | RV | 1 | 0 | 0 | 2 | 0 | Survival/23.4 |
| 7 | 3 | female | 3.73 | LV | 0 | 0 | 0 | 2 | 0 | Survival/19.3 |
| 8 | 70 | female | 1.5 | LV | 0 | 0 | 0 | 1 | 0 | Survival/48.8 |
| 9 | 66 | male | 1.8 | LV | 1 | 0 | 0 | 0 | 0 | Survival/43.9 |
| 10 | 50 | female | 5.33 | LA | 1 | 0 | 0 | 1 | 0 | Survival/37.4 |
| 11 | 68 | female | 4.36 | LA | 1 | 0 | 0 | 2 | 0 | Survival/27.7 |
| 12 | 59 | female | 3.5 | LV | 0 | 1 | 0 | 2 | 0 | Survival/21.7 |
| 13 | 30 | female | 3.3 | RA | 0 | 0 | 0 | 2 | 0 | Survival/29.5 |
| 14 | 47 | male | 2.9 | LV | 1 | 0 | 0 | 0 | 0 | Survival/35.2 |
| 15 | 64 | female | 4.29 | RA | 0 | 1 | 1 | 0 | 1 | Survival/48.1 |
| 16 | 60 | male | 2.57 | RA | 0 | 1 | 1 | 0 | 1 | Survival/44.7 |
| 17 | 42 | male | 3.2 | RV | 1 | 0 | 0 | 0 | 0 | Survival/29.2 |
| 18 | 55 | female | 7.24 | LA | 1 | 1 | 1 | 1 | 2 | 2.7 |
| 19 | 16 | female | 6.5 | RA | 0 | 1 | 1 | 1 | 1 | 4.0 |
| 20 | 33 | female | 1.48 | RV | 1 | 1 | 1 | 1 | 1 | 30.5 |
| 21 | 50 | female | 4.4 | RA | 0 | 1 | 1 | 2 | 1 | 4.7 |
| 22 | 66 | female | 7.74 | RA | 0 | 0 | 1 | 2 | 0 | 16.0 |
| 23 | 30 | male | 7.5 | LA | 0 | 1 | 0 | 2 | 1 | 5.1 |

| AFP (μg/L) | CEA  (μg/L) | CA19-9  (U/ml) | CA125  (U/ml) | T1 TSE  (hypo:0;iso:  1;hyper:2) | T2 TSE  (hypo:0;iso:1;  hyper:2) | PET CT  (negative:0;  positive:1;  null:do not have) | Extracardiac metastasis  (yes:1;no:2) | Diagnosis | benign /malignant |
| --- | --- | --- | --- | --- | --- | --- | --- | --- | --- |
| 2.15 | 1.53 | 0.72 | 8.83 | 1 | 2 |  | 0 | rhabdomyomas | benign |
| 2.29 | 2.64 | 18.8 | 14 | 1 | 2 |  | 0 | rhabdomyomas | benign |
|  |  |  |  | 1 | 2 |  | 0 | rhabdomyomas | benign |
| 1.56 | 0.76 | 6.27 | 11.44 | 1 | 2 |  | 0 | rhabdomyomas | benign |
|  |  |  |  | 1 | 2 |  | 0 | rhabdomyomas | benign |
| 0.72 | 3.05 | 15.02 | 9.4 | 1 | 2 |  | 0 | rhabdomyomas | benign |
| 1.7 | 3.21 | 0.5 | 22.7 | 1 | 0 |  | 0 | fibroids | benign |
|  |  |  |  | 1 | 2 |  | 0 | unclassified tumors | benign |
| 16.4 | 4.5 | 15 | 54.6 | 1 | 2 |  | 0 | unclassified tumors | benign |
|  |  |  |  | 1 | 1 | 0 | 0 | unclassified tumors | benign |
| 2.31 | 2.84 | 16.1 | 19.33 | 1 | 2 | 0 | 0 | unclassified tumors | benign |
| 7.89 | 1.73 | 18.32 | 18.98 | 1 | 1 |  | 0 | unclassified tumors | benign |
|  |  |  |  | 1 | 2 |  | 0 | unclassified tumors | benign |
|  |  |  |  | 1 | 2 |  | 0 | unclassified tumors | benign |
| 0.71 | 0.1 | 5.7 | 39.9 | 1 | 1 | 0 | 0 | unclassified tumors | benign |
| 1.69 | 0.38 | 1.69 | 125.5 | 1 | 1 |  | 0 | unclassified tumors | benign |
|  |  |  |  | 1 | 2 |  | 0 | unclassified tumors | benign |
| 2.02 | 0.48 | 25.23 | 50.02 | 1 | 1 | 1 | 1 | unclassified tumors | malignant |
| 2.38 | 1.4 | 12.65 | 26.6 | 1 | 2 | 1 | 1 | unclassified tumors | malignant |
| 1.92 | 3.07 | 11.87 | 530.2 | 1 | 2 | 1 | 0 | unclassified tumors | malignant |
| 38.42 | 0.38 | 7.67 | 603.5 | 1 | 1 | 1 | 0 | unclassified tumors | malignant |
| 2.34 | 0.94 | 7.05 | 115.2 | 1 | 2 | 1 | 0 | unclassified tumors | malignant |
| 2.79 | 1.27 | 6.62 | 274.3 | 2 | 2 | 1 | 0 | unclassified tumors | malignant |

Abbreviations: LA, left atrium; RA, right atrium; LV, left ventricle; RV, right ventricle; activity, yes,1, no 0; AFP, alpha-fetoprotein; CEA, carcinoembryonic antigen; CA19-9, Cancer Antigens 19-9; CA125, Cancer Antigens 125; PET-CT, Positron Emission Tomography-Computed Tomography.

Table S3. the intra- observer reproducibility of the measurement of quantitative tissue characteristic parameters.

|  | Bias | 95% LOA | CoV(%) | ICC |
| --- | --- | --- | --- | --- |
| **Intra-observer** |  |  |  |  |
| T1 mapping (pre-contrast) | 4.0 | (-20.4 – 28.3) | 0.7 | 0.9859 |
| T1 mapping (post-contrast) | 4.7 | (-32.1 – 41.5) | 2.3 | 0.9769 |
| T2 mapping | 0.19 | (-0.91 – 1.29) | 1.0 | 0.9796 |
| ECV | 0.1 | (-1.9 – 2.2) | 2.0 | 0.9757 |
| **Inter-observer** |  |  |  |  |
| T1 mapping | -1.0 | (-52.6 – 50.7) | 1.5 | 0.9345 |
| T1 mapping (post-contrast) | 1.9 | (-44.0 – 47.8) | 3.0 | 0.9648 |
| T2 mapping | 0.15 | (-1.45 – 1.75) | 1.4 | 0.9563 |
| ECV | 0.2 | (-2.3 – 2.8) | 2.7 | 0.9611 |

Abbreviation: 95% LOA, limits of agreement; CoV, coefficient of variation; SD, standard derivation; ICC, intraclass correlation coefficients; other same as in Table 1.

Reference

1. Yue, P. *et al.* Differential and prognostic value of cardiovascular magnetic resonance derived scoring algorithm in cardiac tumors. *Int. J. Cardiol.* **331**, 281–288 (2021).

2. Li, W. *et al.* Reference value of left and right atrial size and phasic function by SSFP CMR at 3.0 T in healthy Chinese adults. *Sci. Rep.* **7**, 1–11 (2017).

3. Dong, Y. *et al.* Age and Gender Impact the Measurement of Myocardial Interstitial Fibrosis in a Healthy Adult Chinese Population : A Cardiac Magnetic Resonance Study. **9**, 1–9 (2018).
